# Field-Scale AMD Remediation: Microbial Community Dynamics and Functional Insights in Biochemical Passive Reactors

**DOI:** 10.1007/s00248-025-02628-8

**Published:** 2025-11-25

**Authors:** Juliana Jurado, Angela Garcia-Vega, Yaneth Vasquez, Marcela Villegas-Plazas, Fabio Roldan

**Affiliations:** 1 Unidad de Saneamiento y Biotecnología Ambiental (USBA), Departamento de Biología, Pontificia Universidad Javeriana, Cra. 7 No. 40-62, Bogotá, Colombia; 2 Departamento de Biología, Pontificia Universidad Javeriana, Cra. 7 No 40-62, Bogotá, Colombia; 3 Facultad de Ingeniería y Ciencias Básicas, Clúster en Ciencias y Tecnologías Convergentes, Universidad Central, Cra 5 No 21-38, Bogotá, Colombia

**Keywords:** Acid mine drainage (AMD), Biochemical passive reactor (BPR), Active treatment, Passive treatment, Metagenomics, Sulfate-reducing bacteria (SRB)

## Abstract

Acid mine drainage (AMD) generated during coal mining activities is characterized by low pH, high concentrations of dissolved metals and metalloids, and elevated sulfate levels, all of which significantly impact surrounding ecosystems. Scaling up biochemical passive reactor (BPR) systems represents a promising approach for the in situ bioremediation of AMD. While numerous laboratory-scale studies have described the taxonomic and functional composition of microbial communities in BPRs, typically dominated by (ligno)cellulolytic organisms and sulfate-reducing bacteria (SRB), it remains unclear whether this composition is maintained at the field-pilot scale under environmental conditions. To address this gap, 16S rRNA gene metabarcoding and shotgun metagenomics analyses were performed to characterize the taxonomic and functional diversity of microbial communities in the BPRs within a multi-unit field-pilot system. The results revealed that bioremediation effectiveness was driven by syntrophic interactions among hydrolytic, fermentative, and sulfate-reducing bacteria, aligning with laboratory-scale observations. While community composition shifts altered specific taxa, core operational dynamics remained preserved.

**Supplementary Information:**

The online version contains supplementary material available at 10.1007/s00248-025-02628-8.

## Introduction

During mining activities, sulfide mineral ores oxidize upon contact with oxygen, water, and microorganisms, generating acid mine drainage (AMD) [1]. AMD is characterized by an acid pH (< 4.5), high concentration of dissolved metals, metalloid ions, and sulfates, which adversely affect surrounding ecosystems [2]. It disrupts cell division, induces oxidative stress, and inhibits nutrient transport, causing vegetation loss, soil erosion, and ecosystem imbalances [3, 4].

Both active and passive technologies have been developed to treat AMD. Active systems rely on chemical neutralizers (i.e., calcium carbonate and sodium hydroxide), energy input, and mechanical components such as pumps, mixers, and aerators, ensuring rapid and effective treatment in operational mines [5]. However, these systems generate substantial sludge requiring costly waste management [6]. In contrast, passive systems rely on physical, chemical, and biological processes, offering a more sustainable and cost-effective alternative without the need for external energy inputs [7, 8].

Within passive technologies, biochemical passive reactors (BPRs) consist of packed systems with inorganic and organic substrates referred to as reactive mixture (RM) [9]. Inorganic substrates neutralize AMD and optimize hydraulic parameters, such as permeability and conductivity, to regulate hydraulic retention time (HRT) [10]. Meanwhile, organic substrates serve as the primary carbon source for microbial communities where hydrolytic and fermentative bacteria degrade them (e.g., compost and lignocellulosic waste) into simple carbon compounds, gradually releasing small organic molecules such as volatile fatty acids (VFAs), alcohols, that serve as electron donors for SRBs [11, 12]. Through dissimilatory sulfate reduction, SRB produces reactive sulfide, which binds with metal cations in AMD to precipitate metals as metallic sulfides [13]. However, SRB and methanogens often compete for the same electron donors, particularly acetate (acetoclastic methanogenesis) and H_2_/CO_2_ (hydrogenotrophic methanogenesis) [14]. Anoxic soil and sediment studies show that when sulfate is available, SRB typically outcompete methanogens for acetate and H_2_ because sulfate reduction yields more energy, suppressing methanogenesis until sulfate becomes limiting [15, 16].

Microbial community dynamics play a pivotal role in the efficacy of BPRs. Sulfidogenic passive treatments foster diverse acidophilic microbial communities, including SRB and iron-reducing bacteria [17–19], whose abundance and richness increase as pH rises, and carbon sources become available [20]. Key taxa involved in the dissimilatory sulfur process span various phyla (e.g., *Pseudomonadota*, *Bacteriodetes*, *Bacillota*, and *Thermodesulfobacterota*) and genera (e.g., *Chlorobium*, *Devosia*, *Desulfobacter*, and *Desulfomonile*) [18, 21].

Various studies, both in laboratory and field scales, have demonstrated the ability of passive systems to effectively reduce sulfates from AMD, increase pH, and precipitate metals (Co, Cu, Fe, Mn, Ni, and Zn) as metallic sulfide. Bacterial communities are largely dominated by *Bacteroidetes*, *Pseudomonadota*, and *Bacillota* phyla, where *Geobacter* and *Desulfovibrio* have been identified as the main genera responsible for sulfate reduction and metal precipitation [22, 23]. Furthermore, the use of a complex substrate, such as sugarcane vinasse, as an electron donor has been associated with higher microbial diversity compared to simpler electron donors like glycerol and lactate [24, 25].

Metagenomic studies show that passive AMD systems (biochemical reactors, wetlands, BDAS) shift communities from acidophilic, metal-tolerant taxa (e.g., *Acidithiobacillus*, *Leptospirillum*, *Ferrovum*) toward more diverse, functionally redundant consortia enriched in polymer degraders, fermenters, and sulfate-reducing bacteria that underpin metal immobilization and sulfate attenuation [26, 27]. Metagenomic sequencing has identified key profiles, such as sulfate-reducing and iron-oxidizing bacteria, that drive the removal of metals and sulfate, while also highlighting the influence of environmental factors like pH and iron concentration on community structure and function [23, 28]. Shotgun data consistently reveal co-selection of key functions: two-component regulators, ABC metal/ion transporters, cellulose degradation, dicarboxylic-acid pathways, and sulfite/sulfate reduction, supporting resilience and treatment efficiency [27, 29]. Community structure and function track hydrochemical gradients (pH, Fe, SO_4_^2-^), with downstream increases in pH and improved water quality often linked to heterotrophy and biofilm formation [30, 31].

Building on prior research, a laboratory-scale study with synthetic ADM (7 L) established an optimized RM composition for AMD treatment [32]. This mixture supported microbial hydrolysis, fermentation, and sulfate reduction, with dominant taxa including *Pseudomonadota*, *Bacteroidetes*, and *Firmicutes* [11, 22]. Functional profiles revealed significant roles for cellulose degradation, CO_2_ fixation, and metal resistance [33]. To evaluate scalability, this treatment was upscaled to a multi-unit field-pilot system (220 L) in the Zipaquirá mining district (Samacá, Colombia), utilizing both open and closed BPR configurations. Closed reactors outperformed open ones in AMD treatment due to their ability to maintain anaerobic conditions (higher pH/alkalinity, higher SO_4_^2-^ and Fe/Mn removal, critical for SRB activity (lower O_2_ ingress → more negative ORP → DSR favored.

Despite these advances, limited information exists on microbial community composition and functional dynamics after a scaling process such as a multi-unit field-pilot system treating AMD. Comparisons with laboratory-scale columns allow the identification of how scale-up conditions affect microbial community dynamics. Using 16S rRNA gene metabarcoding and shotgun metagenomics, the effects of reactor type, operational time, and reactor section on microbial community dynamics and its correlation with physicochemical parameters were investigated.

## Materials and Methods

### Sample Source

**Fig. 1:**
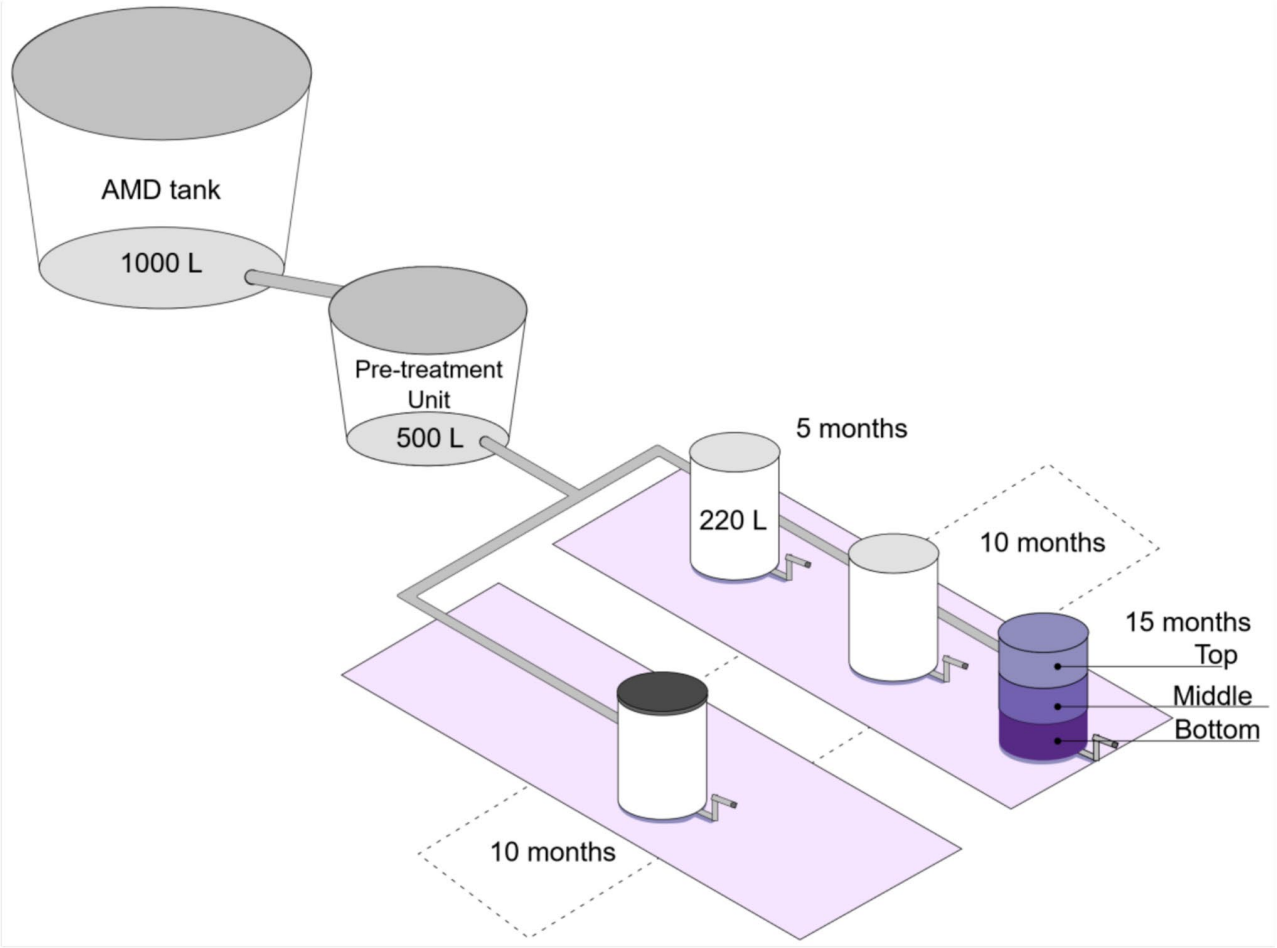
Passive multi-unit field-pilot system for the remediation of the acid mine drainage (modified from Vasquez et al. 2022). Reactor type (open reactors have a gray lid, closed reactors have a black lid), operational time (5, 10, 15 months), and reactor section (top, middle, bottom) are shown

A down-flow, passive multi-unit field pilot was implemented to treat AMD at the Milpa-2 active coal mining site using packed-bed biochemical passive reactors (BPRs) in open and closed configurations, preceded by a dispersed alkaline substrate (DAS) pre-treatment (Fig. 1). The reactive mixture (RM) combined fast- and slow-release carbon with mineral buffering and inoculum. The system was run at HRT ≈ 2 days, following prior lab guidance [9, 31, 32]. For further details, see supplementary material Sect. 1 (Materials and methods).

For this study, sampling commenced once the pilot displayed repeatable performance (≥ 3 consecutive samplings with < 10% variation in pH, alkalinity, SO_4_^2-^, Fe, Mn), where pH increased to ~ 7.6 with sustained alkalinity, and sulfate removal reached 74% (open) and 91% (closed). Iron removal was 63% (open) and 80% (closed), and manganese removal was 48% (open) and 66% (closed).

To estimate initial microbial communities, three samples of the reactive mixture (RM) were taken before BPR start-up. Open BPRs were sacrificed at 5, 10, and 15 months, whereas closed BPRs were sacrificed at 10 months. Following reactor sacrifices, three post-treatment RM samples were collected from each reactor section: top (0–20 cm), medium (20–40 cm), and bottom (40–60 cm) for a total of 39 samples (4 BPR × 3 cores × 3 replicates), including the initial RM as controls. Samples were kept at − 20 °C until processing. All 39 samples underwent 16S rRNA gene metabarcoding, and shotgun metagenomics was performed on the three depth sections of one 10-month closed reactor to resolve depth-wise functional potential under stabilized, low-redox conditions.

### DNA Extraction and Sequencing

To mitigate DNA extraction inhibition by iron precipitates, samples were pre-washed with ammonium oxalate and then rinsed in TE buffer prior to extraction. DNA was extracted (NucleoSpin Soil, SL1 + Enhancer SX) and sequenced on an Illumina NovaSeq [34]. For 16S rRNA metabarcoding, we used 515F/806R (paired-end 2 × 150 bp); shotgun metagenomes were generated from pooled section replicates. Other DNA extraction and sequencing details are provided in the supplementary material.

### Bioinformatics and Statistical Analysis for 16S rRNA gene Metabarcoding

Reads were processed in QIIME 2 with Deblur to infer ASVs and taxonomically assigned with SILVA 138 (515F/806R). Diversity analyses (alpha/beta) were performed after rarefaction in QIIME 2/R, with PERMANOVA for group differences and Kruskal–Wallis/Mann–Whitney with FDR correction for pairwise tests.

### Metagenome Assembly and MAG Annotation

Shotgun reads were quality-filtered (Trimmomatic/FastQC), assembled (metaSPAdes), and binned (MaxBin2, MetaBAT2, CONCOCT) with DAS Tool dereplication. CheckM filtered medium-to-high-quality MAGs; functions were annotated with RASTtk/DRAM and taxonomy assigned with GTDB-Tk following the KBase genome-resolved workflow [35]. The pipeline used in this study is available on the supplementary material  as a static narrative.

### Diversity Correlation with Physicochemical Parameters

Physicochemical parameters, including pH, humidity (%), total organic carbon (TOC), soluble sulfate (SO_4_^2-^, mg Kg^−1^), and metal concentrations (Fe, Mn, Zn, mg Kg^−1^), were measured for samples from open reactors at 5 and 10 months. For final sacrifices (10 and 15 months for closed and open reactors, respectively), only pH, humidity, and TOC were evaluated. Correlation matrices between physicochemical parameters and alpha diversity indices were calculated using the Corplot library in R v4.3.1, and significant values (*p* < 0.05) were visualized.

## Results

In this study, 16S rRNA gene metabarcoding and shotgun metagenomics were used to assess the taxonomical and functional profile of microbial communities in BPRs within a passive multi-unit field-pilot system treating AMD. The variables analyzed included operational time (5, 10, and 15 months), reactor type (open and closed), and reactor section (top, middle, and bottom). Initial microbial communities were estimated from RM.

### Taxonomic Classification of Microbial Communities

**Fig. 2:**
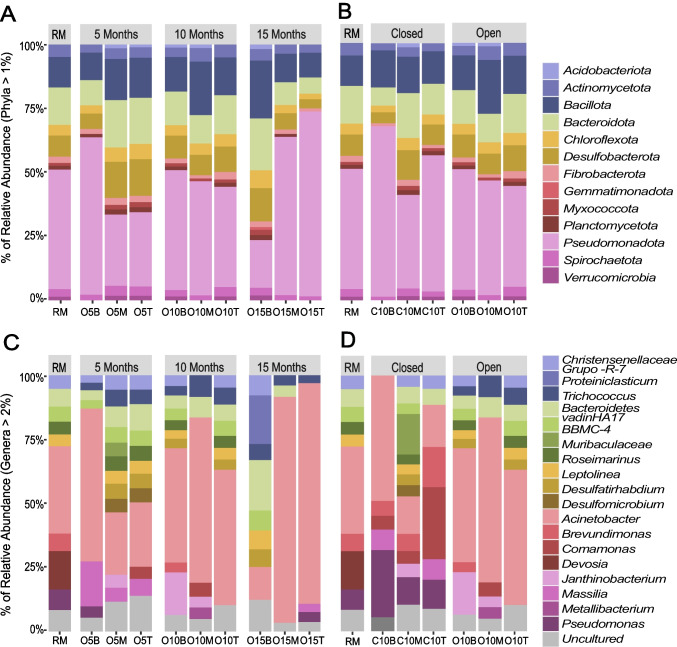
Relative abundance (RA) of phyla and genera across BPRs. **A** and **C** Open BPRs at 0, 5, 10, and 15 months. **B** and **D** Open and closed BPRs at 10 months. X-axis labels denote reactor type (RM = reactive mixture, O = open, C = closed), time (5, 10, 15 months), and section (B = bottom, M = middle, T = top)

The phylum *Pseudomonadota* was the most abundant across all bioreactors, followed by *Bacteroidota*, *Bacillota*, *Desulfobacterota, Actinobacteriota*, and *Chloroflexota* (Fig. 2A and B). Among genera, *Acinetobacter* was the most abundant in both reactor types over time. Other identified genera, including *Brevundimonas*, *Comamonas*, *Pseudomonas*, *Janthinobacterium*, *Masssilia*, *Muribaculaceae*, and *Proteiniclasticum*, exhibited relative abundance (RA) shifts depending on the operational time, reactor type, and section (Fig. 2C and D).

In open reactors, the RA of *Pseudomonadota* peaked in the bottom section at 5 months, but declined over time, whereas the middle and top sections showed increasing RA compared to the reactive mixture (RM) (Fig. 2A). In closed reactors at 10 months, the bottom section had a higher RA of *Pseudomonadota* but a lower RA of *Bacteroidota* and *Chloroflexota*, while the top and middle sections showed an opposite trend (Fig. 2B).

Most of the RA for *Pseudomonadota* was attributed to *Acinetobacter*, with additional contributions from *Brevundimonas*, *Comamonas*, *Devosia*, *Janthinobacterium*, *Massilia*, *Metallibacterium*, and *Pseudomonas* (see Supplementary Material Table 1 for phyla and genus correspondence). In open reactors at 5 months, the bottom section showed higher RA for *Acinetobacter* and *Massilia*, but lower RA for *Bacteroidetes_vadiHA17*, a pattern reversed in the middle and top sections. Over time, *Acinetobacter* populations decreased in the bottom section as *Bacteroidetes_vadiHA17* and *Proteiniclasticum* increased, with an opposite trend in both upper sections. The RA of *Desulfobacteria*, particularly *Desulfatirhabdium*, also increased over time in the bottom section (Fig. 2C).

In both reactor types, the RA of *Pseudomonadota* at the phylum level remained consistent, but genera distributions varied by section (Fig. 2D). For example, the RA of *Acinetobacter* and *Janthinobacterium* differed across open reactor sections, while *Brevundimonas* and *Comamonas* showed more pronounced variations in closed reactors.

### Alpha and Beta Diversity Analysis

**Fig. 3:**
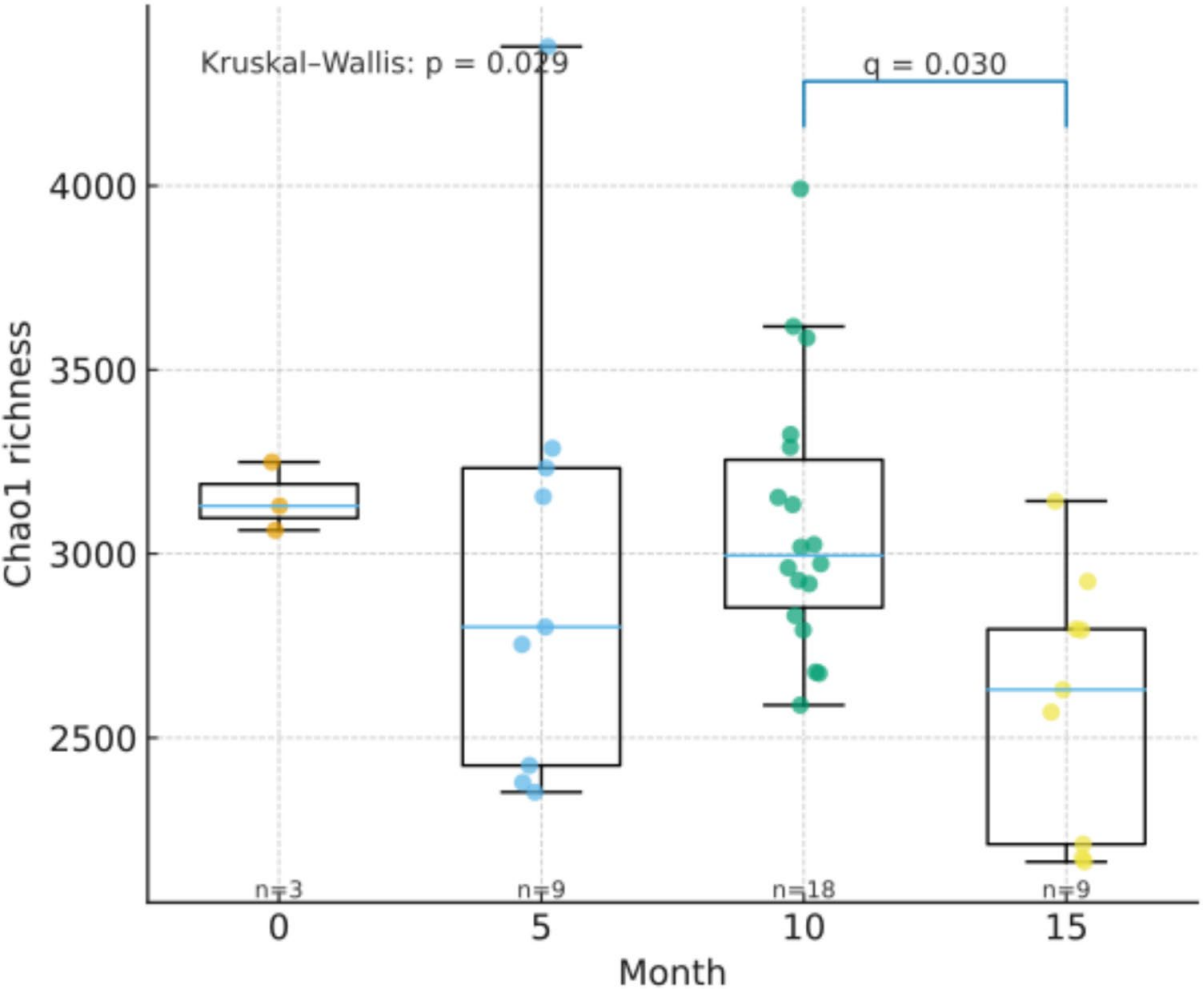
Kluskal-Wallis boxplot of significant differences across operational time

Alpha diversity indices—species richness (observed species and Chao1), evenness (Shannon, Faith, and Pielou’s), and dominance (Simpson)—were calculated for all samples and non-parametric analyses were performed. Only the Chao1 index showed a significant temporal effect on open reactors with a decrease in diversity from 10 to 15 months (*q* < 0.05) (Fig. 3). At 10 months, no alpha index evidenced a detectable effect at the current sample size between Open and Closed reactors. Significant section effects were generally absent; the sole exception was the Shannon index in Closed reactors (KW *p* = 0.027), although pairwise contrasts did not remain significant after FDR correction (small *n* per section). Faith’s PD and Simpson indices did not show robust differences by type or section in these targeted tests. All supporting boxplots (Chao1, Shannon, Faith PD) for the three contrasts and full *p*/*q* tables are provided as Supplementary Fig. S1. No detectable effect at the current sample size.

**Fig. 4:**
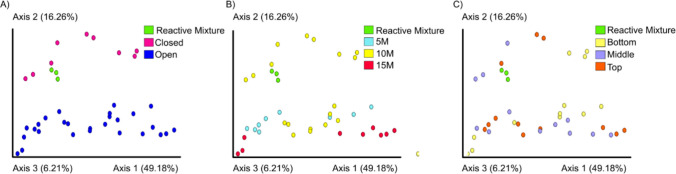
Weighted UniFrac PCoAs. **A** Clusterization by type of BPR (open, closed, and reactive mixture), **B** months of operational time, and **C** section of the reactor

Beta diversity analysis assessed using Weighted UniFrac analysis revealed that reactor type was the main driver of microbial community differences, with operational time also significantly influencing sample clustering (Fig. 4A and B). Reactor sections showed no significant impact on taxonomic profiles (Fig. 4C).

### Functional Gene Prediction of MAGs

**Fig. 5:**
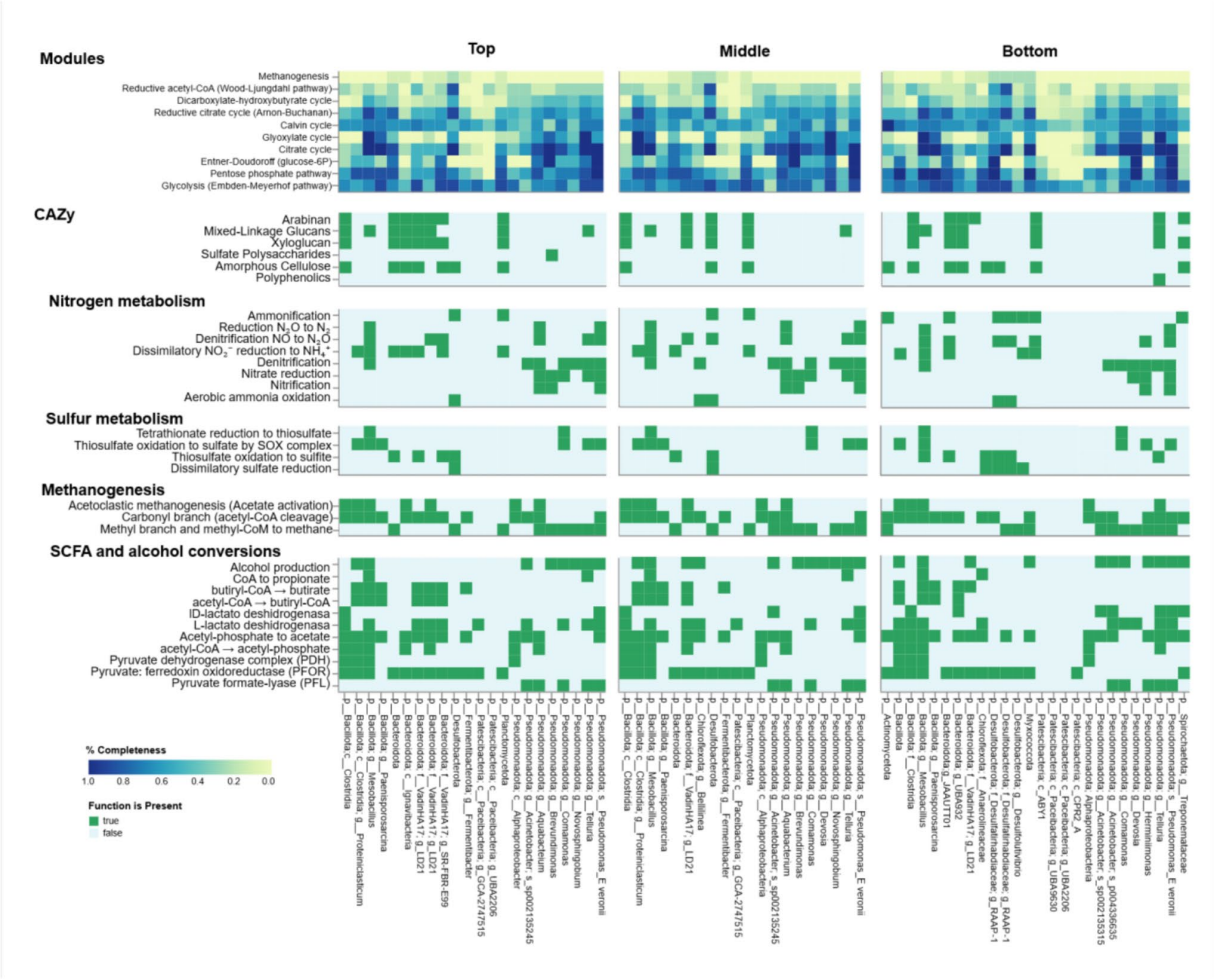
DRAM functional profile of MAGs per section of the 10-month closed reactor

Shotgun metagenomes from the 10-month closed BPRs yielded 22, 20, and 26 MAGs from the top, middle, and bottom sections, respectively. After binning and curation, MAGs were taxonomically assigned and functionally annotated (closest-match assignment ≥ 98% confidence). Across depths, MAGs exhibited broad heterotrophic potential, with near-complete Embden–Meyerhof (EM) and Entner–Doudoroff (ED) glycolytic routes and acetyl-CoA–linked modules (including Wood–Ljungdahl, WL, acetogenic steps). Carbohydrate-active enzymes (CAZy) for xyloglucan, arabinan, mixed-linkage glucans, and amorphous cellulose were common (especially in *Bacteroidota* and *Bacillota/Clostridia* MAGs) supporting polymer hydrolysis and fermentation. Widespread short-chain fatty acid (SCFA) and alcohol conversions (e.g., pyruvate formate-lyase, PFL; pyruvate:ferredoxin oxidoreductase, PFOR; lactate/ethanol dehydrogenases; acetyl-CoA ↔ acetate) indicate community-level redundancy in carbon processing (Fig. 5).

Sulfur and nitrogen metabolisms evidenced a depth-related structure. Sulfur oxidation (SOX) and thiosulfate oxidation occurred in *Pseudomonadota* MAGs in the top/middle, whereas dissimilatory sulfate reduction (DSR; sat–aprAB–dsrAB) was concentrated in *Desulfobacterota* (and some *Bacillota*) MAGs in the middle/bottom, indicating that SRB are the principal terminal electron sinks. *Chloroflexota* MAGs encoded fermentative/acetogenic pathways and dissimilatory nitrate/nitrite reduction to ammonium (DNRA), consistent with syntrophic hand-off to SRB. No MAGs classified as *Archaea* were recovered; However, in some *Bacillota* and *Pseudomonadota* MAGs methanogenesis (mcr-based) genes were annotated. Both acetoclastic and hydrogenotrophic markers were identified at the contig level only.

Altogether, the MAG profiles support a vertical workflow: top: polymer hydrolysis and partial sulfur oxidation; middle: syntrophic fermentation and onset of DSR; bottom: dominant DSR consistent with accumulation of precipitated metals. This organization matches the expected community profile of sulfidogenic reactors.

### Correlation with Physicochemical Parameters

No significant positive correlations (*p* < 0.05) were observed between pH, humidity, and TOC and alpha diversity indices. However, TOC positively correlates with soluble sulfate (SO_4_). Iron concentration had a positive correlation with richness and evenness indices but a negative correlation with pH. In contrast, manganese (Mn) and zinc (Zn) negatively correlated with all diversity indices. Mn also displayed a negative correlation with iron and a positive correlation with Zn. Humidity positively correlated with iron but negatively affected Pielou’s evenness and Simpson indices (SF1). Overall, higher diversity occurs in samples with lower Mn/Zn, higher pH, and moderate moisture, while acknowledging these are correlations.

## Discussion

This study characterized the taxonomical and functional profile of microbial communities within BPRs during AMD treatment in a multi-unit field pilot. Community variations were analyzed across operational time (0, 5, 10, and 15 months), reactor type (open or closed), and reactor section (top, middle, or bottom), while also considering physicochemical parameters. The three sections of the reactor were meant to illustrate the possible stratifications on the 220L reactor as the continuous operation may have caused the vertical flow to influence the distribution of microbial communities and their metabolic functions. Comparison with laboratory-scale columns [29] provided insights into community stratification, with a marked decline in microbial diversity observed over time (0–10 and 10–15 months). This is consistent with progressive selection and functional specialization under stressful bioremediation environments. Furthermore, key metabolic functions associated with contaminant attenuation, such as hydrolysis, fermentation, and sulfur metabolism, were identified.

The persistent dominance of phyla such as *Pseudomonadota*, *Bacteroidetes*, *Bacillota*, and *Thermodesulfobacterota* mirrors the patterns of microbial community stratification observed in laboratory-scale columns, confirming stable reactor performance [9, 25, 36]. The hydrolytic and fermenting capabilities of these taxa suggest a synergistic role with SRB and complex organic compound degraders [37, 38]. However, community composition differed between laboratory and field-pilot systems, likely due to contrasting conditions. The field pilot was exposed to the elements and had a downwards flow, while the laboratory columns had synthetic AMD exposure and an upwards flow; also, they had different inoculum sources.

In laboratory-scale columns, *Treponema* was the most abundant genus, likely due to its fermentative role and inoculum composition. In contrast, *Acinetobacter* dominated the field pilot system, possibly owing to its metabolic versatility, including broad-substrate utilization and heavy metal tolerance under acidic conditions [39]. The significant negative correlation (*p* < 0.05) between alpha diversity and manganese (Mn) concentrations suggests *Acinetobacter*’s involvement in Mn-dependent processes, such as autotrophic denitrification and anaerobic iron oxidation [40]. Its peak dominance at 5 months coincides with elevated Mn levels, particularly in bottom sections. Moreover, the field system’s iron (Fe) concentrations were > 1000-fold higher than in lab columns, consistent with *Acinetobacter*’s prevalence in iron-rich environments [41].

Functional roles also varied between the systems. In laboratory-scale BPRs, hydrolytic functions were attributed to the genera *Devosia*, *Clostridium*, *Bacteoides*, *Sphingobacterium*, and *Sphingomonas* while fermenting roles were assigned to *Brevundimonas*, *Treponema*, and *Agrobacterium*. In the field pilot, the distribution of these tasks was different depending on the configuration of the reactor. In the closed reactors, *Acinetobacter* was less abundant and other *Pseudomonadota* genera like *Brevundimonas*, *Comamonas*, *Pseudomonas*, *Janthinobacterium*, and *Massilia* seemed to be taken on hydrolytic and fermentative roles [42]. Additionally, anaerobic genera like *Muribaculaceae* and *Proteiniclasticum* contributed cellulolytic and fermentative functions, reflecting the adaptability of microbial communities to reactor configurations [43, 44].

The clear differences in treatment efficiency and mechanisms are reflected in the microbial communities. The results of the previous study on the solid waste post-treatment evidenced that the ratio AVS/SEM of open BPRs (1.8 × 103) was 400 times greater than that of the ratio of closed BPRs (3.9 × 101). Therefore, the precipitation of oxy-hydroxide and carbonate minerals was the predominant mechanism of removing metals in open configurations, while the formation of metal sulfides was predominant in the closed ones. These findings indicate that, in PBRs-A, metals (Fe2 +, Mn2 +, and Zn2 +) persisted in the interstitial water were not immobilized in the reactive mixture and leached to the environment. The different precipitation mechanisms reflected on the community composition, with the reactor type being the variable that clustered the data the most on the Weighted UniFrac beta diversity analysis [45].

With both metabarcoding and MAGs reconstruction, SRBs were found in all samples. This SRB activity was associated with high diversity and abundance of hydrolytic and fermentative bacteria, which ensured a steady supply of electron donors [46]. These findings highlight the importance of maintaining both simple and complex carbon sources to support microbial community longevity and performance. Diverse microbial communities enhance hydrolysis and fermentation, ensuring electron donors for SRBs. Simple carbon sources sustain microbial activity, while complex polymers provide a long-term carbon reservoir [47].

The presence of methanogenesis markers without an Achaea MAG could reside in (i) low-abundance or fragmented archaeal populations whose contigs assembled yet remained unbinned and (ii) a minor possibility of cross-binning (archaeal scaffolds placed into bacterial bins) or over-calling by profile HMMs at lenient cutoffs. Accordingly, while methanogenesis genes occur in the metagenome, the binning did not recover archaeal genomes, so we do not assign methane production to specific MAGs. Read-mapping profiles nevertheless indicate that methanogenesis potential is enriched in deeper sections, aligning with reducing conditions in closed BPRs [15]. Aligned with the known competition/partitioning between SRB and methanogens as sulfate becomes locally depleted.

A recent genome-resolved study of AMD-impacted mine tailings reported clear depth–related patterns in community structure and function. The Calvin Cycle, WL pathway, and rTCA cycle were the main carbon-fixing pathways. These were correlated with the high relative abundances of potential carbon fixers, where *Actinobacteria* exhibited the greatest enrichment [48]. Consistently, MAGs from the present system recovered the same carbon-fixation pathways, and the metabarcoding likewise indicated a high relative abundance of *Actinobacteria*. Additionally, SOX systems were relatively abundant in surface tailings, and *Deltaproteobacteria* were the most prevalent group [48]. In our system, an analogous role is fulfilled by *Pseudomonadota*. The same study found that 45 MAGs encoded at least one copy of *dsrA*, with higher total relative abundance in deeper tailings, mirroring the enrichment of dissimilatory sulfate reduction under more reducing conditions [48]. Consistently, an up-flow anaerobic packed-bed reactor showed the same vertical stratification: fermenters dominated the inlet zone (evidenced by propionate accumulation), and SRB consumed these fermentation products to reduce sulfate downstream [49]. Complementary work testing organic-carbon sources and inoculum in sulfate-reducing bioreactors demonstrates that donor quality and mixtures strongly govern long-term SO_4_^2-^ and Fe removal consistent with the fermenter to SRB hand-off observed at the genome level [50]. Together, these findings support a genome-informed design principle: optimize sealing and donor regimes to reinforce carbon hydrolysis, fermentation, and DSR cascade, stabilize low-redox niches, and maximize sulfide-mediated metal capture.

### Study Limitations and Perspectives

This study integrates 16S rRNA gene metabarcoding and shotgun metagenomics to study BPR microbial ecology; however, there were several constraints for the interpretation. First, the metagenomic dataset was limited in sample size and scope (genome-resolved analyses were restricted to one closed reactor at a single time point), which reduces statistical power to resolve reactor type and temporal effects. Second, configurations and time replication were uneven between methods: all sections were profiled by 16S, whereas metagenomes were not. And third, the physicochemical parameters were also not measured for all samples that had a metabarcoding analysis, limiting the scope and the possibility to draw fundamental conclusions.

Future work should (i) expand replicated, time-series metagenomes across reactor types and depths; (ii) incorporate metatranscriptomics/proteomics/metabolomics to connect potential to activity; and (iii) apply genome-resolved and differential-coverage analyses to improve MAG recovery and strain-level dynamics. This integrated design will more robustly link community structure, function, and performance in field BPRs.

## Conclusion

Overall, these studies indicate that the metabolic route for AMD bioremediation is well established, as it followed the same principle of syntrophic interactions among hydrolytic microorganisms, fermenters, and sulfate-reducing bacteria previously observed in the laboratory-scale columns. Yet rigorous cross-system comparisons during scale-up remain necessary. As here was revealed the importance of ensuring a more anaerobic environment with a closed operation. Such comparisons should test how field-relevant variables (hydraulics, sealing, electron-donor supply, and mineralogy) shape microbial diversity and function, crucial to ensuring the highest treatment efficiency set-up.

## Supplementary Information

Below is the link to the electronic supplementary material.

ESM 1 (XLSX 75.2 KB)

ESM 2 (DOCX 2.17 MB)

## Data Availability

16S rRNA gene metabarcoding and shotgun metagenomics raw data was deposited in the NCBI under the project accession number PRJNA1263004.
