# Supplementary material for: Field-Scale AMD Remediation: Microbial Community Dynamics and Functional Insights in Biochemical Passive Reactors
Source: Microb Ecol. 2025 Nov 25;89(1):8. doi: 10.1007/s00248-025-02628-8 (PMC12764544; doi:10.1007/s00248-025-02628-8)
Supplement: Supplementary file 2 — (DOCX 2.17 MB) [file 248_2025_2628_MOESM2_ESM.docx]

**Section1: Materials and methods**

**Sample Source**

A down-flow, passive multi-unit field pilot (Figure 1) was built to treat AMD at the Milpa-2 active coal-mining site (5°27’59’’ N; 73°33’23’’ W) in the Zipaquirá mining district (Samacá, Colombia). Each packed-bed biochemical passive reactor (BPR; ~220 L; ~80 cm height × ~60 cm diameter) was assembled bottom-up with 10 cm of gravel (Ø≈1 cm), a nylon mesh (≈70 µm), a reactive mixture layer (155–180 L packed in ~15 cm lifts), a second mesh, and a 10 cm gravel cap. Two configurations were evaluated: open BPRs with a woodchip cover allowing gas exchange, and closed BPRs sealed with an airtight lid and gasket to lower O₂ ingress and maintain more negative redox potential. Upstream, a dispersed alkaline substrate (DAS) pretreatment unit (~500 L; wood ash:woodchips 1:1 over a perforated manifold) buffered acidity and captured ferric oxides/hydroxides before the BPRs. The reactive mixture was selected from prior optimization work to balance rapid and long-term carbon delivery and permeability, and consisted of 15% cow manure, 10% mushroom compost, 25% sawdust, 20% gravel, 15% limestone, and 15% AMD-acclimated sediment inoculum (v/v, packed). This blend provides C:N appropriate to microbial needs, hydraulic conductivity in the range k ≈ 4.5×10⁻² cm s⁻¹; porosity ≈ 0.49–0.53, supporting homogeneous flow and sulfate-reducing conditions (start-up inoculation with wetland sediment). The system operated at a hydraulic retention time (HRT) of 2 days, verified weekly by volumetric checks; reactors were saturated and acclimated for ~2 weeks prior to continuous operation (reactor design, mixture rationale, and hydraulics following field and lab guidance in Vásquez et al., 2016a; 2016b; 2018; 2020; 2022).

**DNA Extraction and Sequencing**

To eliminate precipitated metals (primarily iron) that inhibited DNA extraction, samples underwent a pre-wash treatment with a 100 mL of 0.3 M ammonium oxalate (pH 3.0) under constant agitation for 20 min [38]. This step was repeated until the supernatant became colorless and free of iron. Subsequently, the samples were washed three times with TE buffer [10 mM Tris-HCl, 1 mM EDTA, pH 8.0] to remove any remaining ammonium oxalate.

DNA was extracted from 1 g samples using the NucleoSpin Soil kit (Macherey-Nagel) with SL1 buffer and 10 µL Enhancer SX, following the manufacturer’s protocol. Sequencing for each analysis was performed using the Illumina NovaSeq (Illumina, USA).

For 16S rRNA gene metabarcoding a paired-end 2x150 pb library was prepared using universal primers 515F (5’ GTGCCAGCMGCCGCGGTAA 3’) – 806R (5’ GGACTACHVGGGTWTCTAAT 3’). Meanwhile, for shotgun metagenomics, DNA extracts (from replicates taken per reactor section) from replicates of each reactor section were pooled for sequencing. Both 16S rRNA gene metabarcoding and shotgun metagenomics raw data was deposited in the NCBI under the project accession number PRJNA1263004.

**Bioinformatics and statistical analysis for 16S rRNA gene metabarcoding:**

Sequence reads were processed in QIIME 2 v1.8 with Deblur to denoise and infer ASVs. After quality inspection, adapters and low-quality tails were trimmed and paired-end reads merged prior to denoising. 7,092 ASVs were obtained and taxonomically assigned with a pre-trained SILVA 138 classifier for the 515F/806R region (99% identity). The phylogeny was inferred with IQ-TREE v2.2.2.3. Rarefaction curves and relative-abundance summaries were produced in R v4.3.1 (phyloseq/ggplot2). For diversity analyses, the feature table was rarefied to 1,000 sequences/sample. Alpha metrics (Observed features, Chao1, Shannon, Pielou’s evenness, Faith’s PD, Simpson) were computed in QIIME 2 and compared using Kruskal–Wallis with Mann-Whitney U Test pairwise and Benjamini–Hochberg FDR where applicable. We performed three prespecified contrasts: (i) BPR type (Open *vs.* Closed) at 10 months; (ii) time within Open reactors (5, 10 and 15 months); and (iii) section within each reactor type (Top/Middle/Bottom). Beta diversity (Jaccard, Bray–Curtis, unweighted/weighted UniFrac) was visualized by PCoA; group differences were assessed by PERMANOVA (999 permutations). Statistical significance was set at p (or q) < 0.05.

**Metagenome Assembly and Gene** Annotation

Raw metagenomic reads were processed on the KBase platform: trimming with Trimmomatic v0.36 (LEADING:3, TRAILING:3, SLIDINGWINDOW:4:15, MINLEN:36) retained high-quality data as verified with FastQC v0.11.9 (Q30 metrics), yielding per-sample survival of 22,345,197/22,554,659 reads for AT (99.07%), 25,520,810/25,793,448 for AM (98.94%), and 27,697,854/27,924,068 for AB (99.19%). Each sample was assembled independently using metaSPAdes v3.15.3, MEGAHIT v1.2.9, and IDBA-UD v1.1.3, retaining contigs ≥300 bp [40]; assemblies were compared in KBase (Compare Assembled Contigs v1.1.2), and metaSPAdes outputs were selected for downstream analyses based on superior statistics (e.g., largest contig >772 kb; 90,657 total contigs). From each sample’s selected assembly, metagenome-assembled genomes (MAGs) were reconstructed using multi-tool binning with MaxBin2 v2.2.4, MetaBAT2 v1.7, and CONCOCT v1.1, followed by optimization and de-replication with DAS Tool v1.1.2 to obtain a non-redundant bin set. Genome quality was assessed with CheckM v1.0.18, retaining only medium-to-high-quality MAGs (completeness ≥50%, contamination ≤10%). Retained MAGs were functionally annotated with RASTtk v1.073 (default parameters; Bacteria domain), taxonomically classified with GTDB-Tk v2.3.2 (default settings), and their metabolic potential summarized and visualized with DRAM. The Kbase narrative was based on Chivian et al. 2023 method on metagenome-assembled genome extraction and analysis. The pipeline used on this study is available here as an [static narrative](https://kbase.us/n/135958/162/) (https://kbase.us/n/135958/162/).

**Section 2: Results**

**Alpha and Beta Diversity analysis**

**
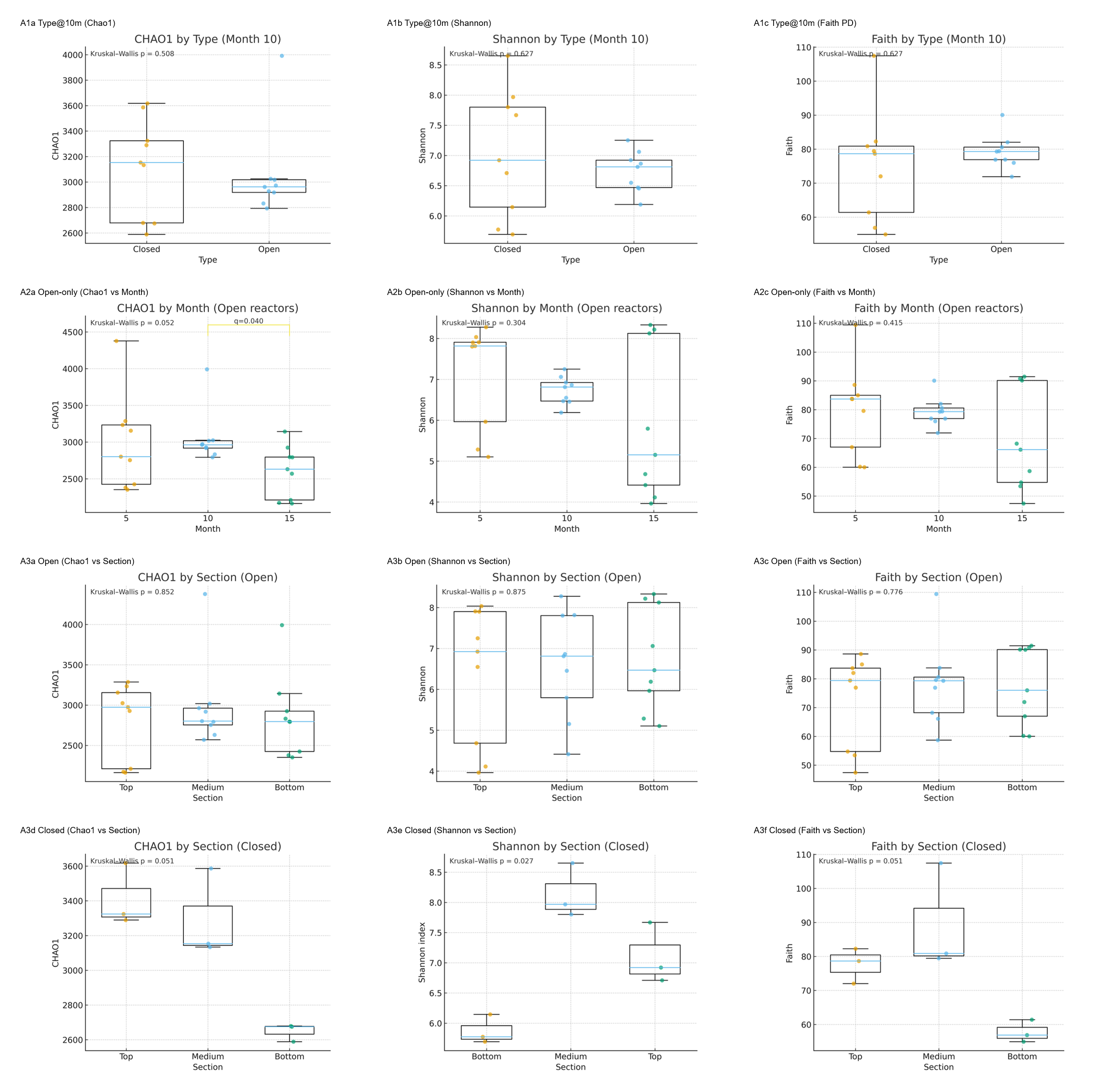
**

**Figure S2.** Kluskal-Wallis boxplots of all group comparisons.

**Taxonomic classification of microbial communities:**

**Table 1**. Genus–phylum correspondence

| Phyla | Genera |
| --- | --- |
| *Bacillota* | *Bacillus* |
|  | *Christensenellaceae Grupo -R-7* |
|  | *Proteiniclasticum* |
|  | *Trichococcus* |
| *Bacteroidota* | *Bacteroidetes vadinHA17* |
|  | *BBMC-4* |
|  | *Muribaculaceae* |
|  | *Roseimarinus* |
| *Chloroflexotaexota* | *Leptolinea* |
| *Desulfobacterota* | *Desulfatirhabdium* |
|  | *Desulfomicrobium* |
| *Pseudomonadota* | *Acinetobacter* |
|  | *Brevundimonas* |
|  | *Comamonas* |
|  | *Devosia* |
|  | *Janthinobacterium* |
|  | *Massilia* |
|  | *Metallibacterium* |
|  | *Pseudomonas* |

**Correlation with physicochemical parameters:**


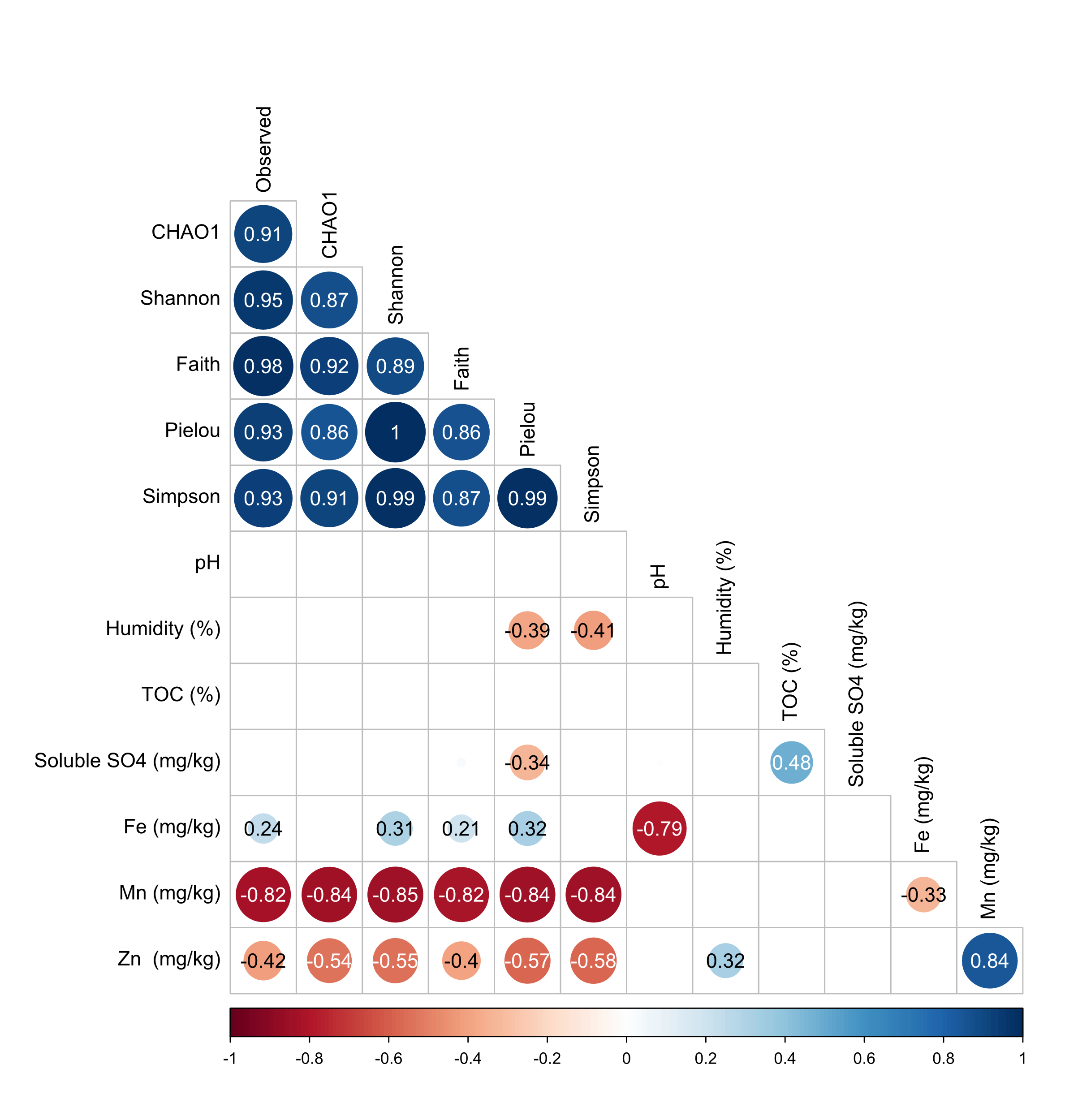


**Figure S2.** Positive correlation plot between the physicochemical parameters (pH, humidity, soluble sulfate, total organic carbon, iron, manganese, and zinc concentrations) and alpha diversity indices of the open BPR at 5 and 10 months.
